# Disulfiram as a Therapeutic Agent for Metastatic Malignant Melanoma—Old Myth or New Logos?

**DOI:** 10.3390/cancers12123538

**Published:** 2020-11-27

**Authors:** Francisco Meraz-Torres, Sarah Plöger, Claus Garbe, Heike Niessner, Tobias Sinnberg

**Affiliations:** 1 Center for Dermatooncology, Department of Dermatology, University Hospital Tübingen, 72076 Tübingen, Germany; francisco.meraz-torres@student.uni-tuebingen.de (F.M.-T.); sarah.ploeger@med.uni-tuebingen.de (S.P.); Claus.Garbe@med.uni-tuebingen.de (C.G.); heike.niessner@med.uni-tuebingen.de (H.N.); 2 Cluster of Excellence iFIT (EXC 2180) Image Guided and Functionally Instructed Tumor Therapies, University Hospital Tübingen, 72076 Tübingen, Germany

**Keywords:** melanoma, disulfiram, systemic anti-cancer therapy

## Abstract

**Simple Summary:**

In recent years, disulfiram has gained in attention as an anticancer drug due to its broad activity against various cancers, and its mechanisms and molecular targets have been deciphered in vitro and in vivo. One of these cancers is melanoma. Initial data from human studies show some benefit, but do not confirm its broad efficacy as a monotherapy. However, combination approaches could pave the way for exploiting the beneficial effects of disulfiram for cancer patients, including those with melanoma.

**Abstract:**

New therapeutic concepts such as anti-PD-1-based immunotherapy or targeted therapy with BRAF and MEK inhibitors have significantly improved the survival of melanoma patients. However, about 20% of patients with targeted therapy and up to 50% with immunotherapies do not respond to their first-line treatment or rapidly develop resistance. In addition, there is no approved targeted therapy for certain subgroups, namely BRAF wild-type melanomas, although they often bear aggressive tumor biology. A repurposing of already approved drugs is a promising strategy to fill this gap, as it will result in comparatively low costs, lower risks and time savings. Disulfiram (DSF), the first drug to treat alcoholism, which received approval from the US Food and Drug Administration more than 60 years ago, is such a drug candidate. There is growing evidence that DSF has great potential for the treatment of various human cancers, including melanoma. Several mechanisms of its antitumor activity have been identified, amongst them the inhibition of the ubiquitin-proteasome system, the induction of reactive oxygen species and various death signaling pathways. This article provides an overview of the application of DSF in humans, its molecular mechanisms and targets in cancer therapy with a focus on melanoma. The results of clinical studies and experimental combination approaches of DSF with various cancer therapies are discussed, with the aim of exploring the potential of DSF in melanoma therapy.

## 1. The History of the Discovery and Development of Disulfiram

Disulfiram (DSF) was first synthesized as a novel composite from thiocarbamide (thiourea) in 1881 by the German chemist M. Grodzki, who also reported its stoichiometric formula C_10_H_2_0N_2_S_4_ [1]. Throughout the next 20 years, several new substances were derived from disulfiram and used mainly for textile processing. In the early 1900s, DSF was used in the European rubber industry to improve and accelerate the vulcanization of rubber such as neoprene. From then on, it served as an industrial catalyst for the development and manufacture of rubber worldwide for over 30 years until the late 1930s [2]. 

In 1937, E.E. Williams reported for the first time on the alcohol intolerance of workers who were exposed to DSF during the vulcanization of rubber and consumed alcohol [3]. The alcohol-adverse symptoms described by E.E. Williams ranged from very mild symptoms such as sweating, hot flushes, shortness of breath or vomiting to severe symptoms as respiratory depression, cardiovascular collapse, seizures and even death [4]. Similar spectacular effects were reported in 1939 from the rubber boot production in Sweden. At that time, however, no further research was conducted regarding disulfiram and its physical effects after alcohol consumption [3]. 

In 1947, a research group at the University of Copenhagen in Denmark led by J. Hald and E. Jacobsen investigated DSF as an antiparasitic therapeutic agent for intestinal worm infections. A vermifuge effect was shown in rabbits. Based on these results, it was also decided to test DSF against verminal infections in humans. Again, alcohol-aversive effects were observed if the patients consumed alcohol. In 1948, it was found and published that the strong interactions of DSF with alcohol are due to an observed accumulation of acetaldehyde. The interactions proved to be dose-dependent in patients using up to 3000 mg of DSF per day [5].

With this knowledge about the alcohol-intolerance-mediating property of DSF, Ruth Fox began administering DSF to treat alcohol addiction in the United States in 1949. Due to the good results and efficacy, the FDA considered DSF as safe and effective. Finally, DSF was approved in 1951 for the treatment of alcohol addiction in the United States. Shortly after the approval, the Wyeth-Ayerst Laboratories began manufacturing Antabuse^®^ tablets [4]. Since then, DSF was studied in various fields such as parasitology, infectious diseases, and oncology. With a median lethal dosage (LD50) of 8.6 g/kg, Antabuse^®^ is very safe and has been used for the management of alcohol dependency for the last 60 years [4,6,7,8,9].

## 2. Chemical Structure of Disulfiram and Its Metabolites

Disulfiram (DSF; CAS number 97-77-8) is a tetraethyl derivate of the organic sulphur-containing dimethyl-dithiocarbamate (tetraethyl thiuram disulfide), with the IUPAC name 1-(diethylthiocarbamoyldisulfanyl)-*N*,*N*-diethylmethanethioamide [10,11]. It is relatively small, with a molecular weight of 296.5 g/mol and the stoichiometric formula C_10_H_20_N_2_S_4_. DSF is a light-grey crystalline powder, with good oral bioavailability and high absorption rates (85–90%) [4,11]. Its main immediate metabolite is the diethyldithiocarbamate (DDC; IUPAC name *N*,*N*-diethylcarbamodithioate), a dithiocarbamate anion resulting from the release of a proton from the diethyldithiocarbamic acid during the acidic cleavage of DSF. DDC has a molecular weight of 148.3 g/mol, with the chemical stoichiometric formula C_5_H_10_NS_2_ [4,10,11].

DSF has been known worldwide as Antabuse^®^ or Antabus^®^ since 1952. This name was given by Jacobsen because of its ability to form a stable dark precipitate when combined with copper (Cu^2+^) and after being recrystallized with carbon tetrachloride to improve its gastrointestinal absorption [4,11]. 

## 3. Pharmacology and Metabolism of Disulfiram

**Figure 1:**
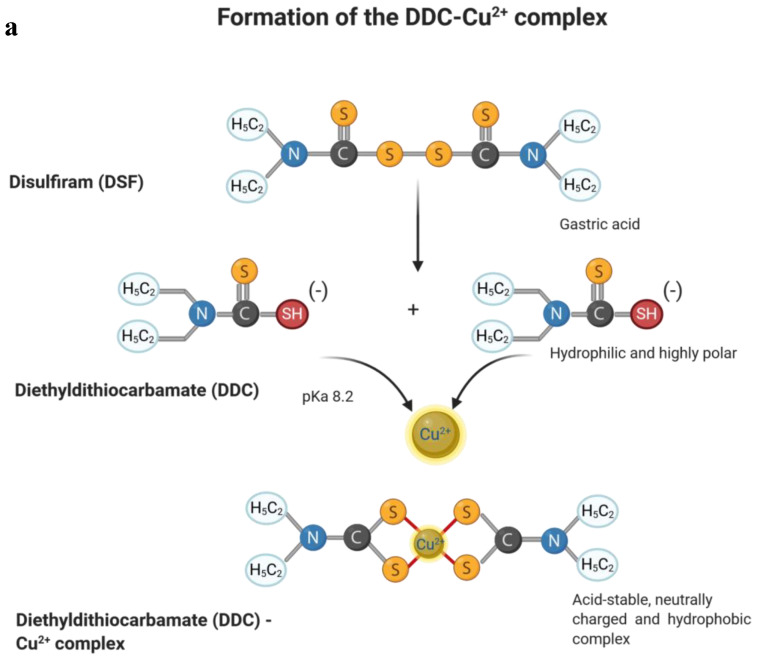

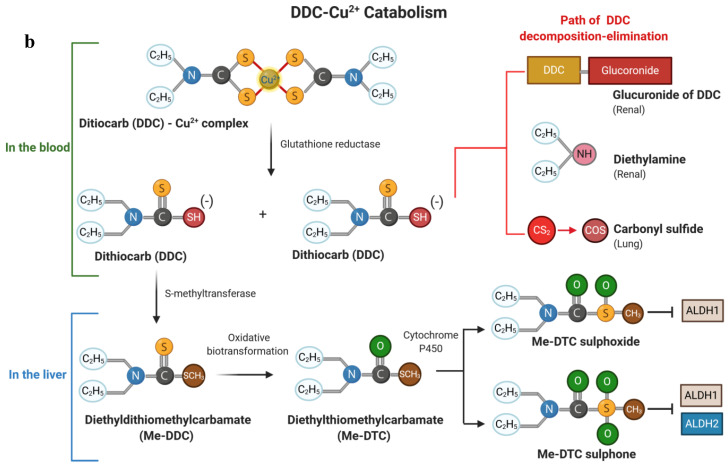
Metabolism of disulfiram (DSF) and its main metabolite diethyldithiocarbamate (DDC). DSF is rapidly hydrolyzed into two molecules of its active metabolite DDC which can complex metal ions like copper (II) (**a**). DDC is further catabolized by several metabolic processes that end up in the inhibition of aldehyde dehydrogenase (ALDH1/2) and different elimination routes (**b**).

After ingestion, 99% of DSF are quickly and irreversibly converted into the corresponding thiol, diethyldithiocarbamate (DDC), due to the low pH of gastric acid in the stomach [4,6,12] (Figure 1a). DSF and DDC, respectively, are easily absorbed by the upper gastrointestinal tract and unaffected by ingestion of food [9,11]. Unabsorbed DDC, which is between 10 and 15%, is excreted in the feces. DDC is a hydrophilic, highly polar compound, which is easily decomposed into carbon disulfide (CS_2_) and diethylamine (DEA) in an acidic environment [4,6,12]. CS_2_ is oxidized to carbonyl sulfide (COS) and can be further oxidized to carbon dioxide, generating sulfur radicals [7,12]. DDC chelates strongly available heavy metal ions, for example, copper (II) (Cu^2+^), forming the bis-(diethyldithiocarbamate)-Cu^2+^ complex (DDC-Cu^2+^) [4,8,9,10,12,13]. DDC-Cu^2+^ is an extremely acid-stable, neutrally charged, and particularly hydrophobic copper complex, which facilitates its absorption along the entire length of the upper gastrointestinal tract [4,6,12]. In this way, 80–95% of the disulfiram administered orally are absorbed and the remaining 5–20% excreted [4,6,11,12]. Accordingly, absorption and further distribution of the DSF via the gastrointestinal mucosa into the blood predominantly involves the DDC-Cu^2+^ complex [4,6,12] (Figure 1b). In the blood, the complex splits into diethyldithiocarbamate monomers through the action of the glutathione reductase system of the erythrocytes [12,14]. DDC reacts with free thiol groups of various proteins, especially albumin, to form mixed disulfides [7,12,14]. Free DDC can be measured in serum only for a short time [7]. DDC is a substrate for phase II metabolism, whereby diethyldithiomethyl-carbamate (Me-DDC) is formed under the influence of S-methyltransferase [12,14]. In the liver, Me-DDC is oxidatively bio-transformed into diethylthiomethylcarbamate (Me-DTC), which is further oxidized to the corresponding sulfoxide and sulfone (S-oxide) metabolites by the microsomal cytochrome P450 monooxygenases [4,12]. These S-oxidant metabolites are strongly involved in the formation of a covalent cysteine adduct with the enzyme aldehyde dehydrogenase (ALDH), and lead to its inhibition [12]. 

DSF metabolites are mainly excreted in the feces, via the kidneys or exhaled by the lungs [12]. About 65% of free DDC in the blood are covalently bound to glucuronic acid and are excreted in urine as glucuronide of the DDC. Remaining DDC is mainly converted into DEA and CS_2_ and excreted or exhaled [7,12].

Me-DTC blocks the activity of the enzyme aldehyde dehydrogenase (ALDH), causing an accumulation of acetaldehyde in the body. Me-DTC acts as a suicide inhibitor for the mitochondrial (low K_m_) ALDH1. It inhibits ALDH1 at concentrations of about 0.1 μg/mL in a dose-dependent manner [4,6,12]. The S-oxidized metabolites, especially the sulfone metabolite, are strong inhibitors of ALDH1 but also the mitochondrial (low K_m_) isozyme ALDH2 [12,14]. As a result, both key isozymes for the removal of acetaldehyde in the process of alcohol oxidation can be efficiently inhibited by DSF [4,6,11,12]. 

ALDH oxidizes ethanol to acetaldehyde in the liver and brain, causing a release of high concentrations of histamine, which is responsible for the alcohol flush reaction [4,12,15]. Acetaldehyde is normally rapidly oxidized to acetate by ALDH2 in the liver, which then enters the tricarboxylic acid cycle [12]. Consequently, the inhibition of ALDH2 by DDC metabolites leads to a high accumulation of acetaldehyde after ethanol consumption, resulting in very unpleasant symptoms known as “disulfiram ethanol reaction” [4,6,11,12,15]. These range from moderate symptoms like nausea, vomiting, flushing of the skin, vasodilatation, tachycardia, tachypnea, breathlessness, palpitation, and headache to severe physiologic reactions like cardiovascular collapse, acute congestive heart failure and death [4,6,11]. However, DDC does not disturb the rate of ethanol elimination [12]. DSF is normally well tolerated by the patients with a low rate of adverse events besides the ethanol-induced reactions [15]. Due to the disulfiram-ethanol reaction, DSF has been used for the treatment of alcohol dependence for more than 60 years [4,6,8,9,10,11,15,16,17].

## 4. In Vitro Activity of Disulfiram against Cancer Cells

### 4.1. Activity against Cancer Cells

Various cancers cell models have been tested for the anticancer cytotoxicity of DSF. It showed strong cytotoxic effects against several cancer cell lines, originating from prostate cancer, breast cancer, nasopharyngeal cancer, non-small cell lung cancer [16] and melanoma [18]. The most commonly used cell viability tests were the alamarBlue and MTS assays [18,19], which determine living metabolically active cells based on their redox status, or a live dead stain (calcein AM/propidium iodide) [19]. It was found that different cell death mechanisms are involved, e.g., the extrinsic apoptosis signaling pathway in melanoma [18], but also ferroptosis in nasopharyngeal carcinoma models [16]. The intrinsic apoptotic pathway did not seem to be involved in the DSF-mediated melanoma cell death [18]. In contrast, DSF has been shown to prevent apoptosis in benign rat thymocytes. [20]. Addition of Cu^2+^ significantly enhanced the DSF-induced inhibition of cancer cell proliferation and viability [9]. Hence, disulfiram was identified to have a broad anticancer effect in vitro, and Cu^2+^-chelation of the principle metabolite diethyldithiocarbamate (DDC or ET) seemed to be crucial for its tumor cell killing activity [9,18,19,21,22]. This cytotoxic activity was promoted by ALDH-independent methods [16]. It was further shown that the copper complex DDC-Cu^2+^/CuET was preferentially accumulated in tumor tissue compared with other tissue, like liver or plasma [9]. 

### 4.2. Activity against Melanoma Cells

Some of the cancer-cell-killing effects of DSF, mentioned above, have been also described in melanoma cells. Disulfiram decreased proliferation and induced apoptosis in melanoma cell lines [18]. The presence of free bivalent metal ions like Cu^2+^ or Zn^2+^ strongly enhanced the anti-proliferative and pro-apoptotic effects of DSF [18]. These effects were measured by either cell proliferation assays (CellTiter 96 AQueous, Promega, Madison, WI, USA) or nuclear apoptotic body/DAPI (4′,6-diamidin-2-phenylindol) stainings. Apoptosis was additionally analyzed by the quantification of outer surface phosphatidyl serine with AnnexinV or by the detection of cleaved caspase 3 [18,22]. Further, it was found that DSF affects both melanoma cells originating from nodular and superficial spreading melanomas [18] as well as melanoma cells from different growth phases, namely the radial, vertical and metastatic growth phases [23]. However, no acral-lentigenous, lentigo-malignant or mucosal melanoma cells have been investigated to date. Metastatic melanoma cell lines (A375, C832C, C8146A, and C8161) were more sensitive to DSF than cell lines derived from primary melanomas [18]. DSF mediated in vitro cytotoxicity was rather specific for melanoma cell lines, as these were more affected by disulfiram than melanocytes and other benign cells [18,23].

## 5. Mode of Action of Disulfiram on Cancer Cells 

### 5.1. Disulfiram and ROS Formation

**Figure 2:**
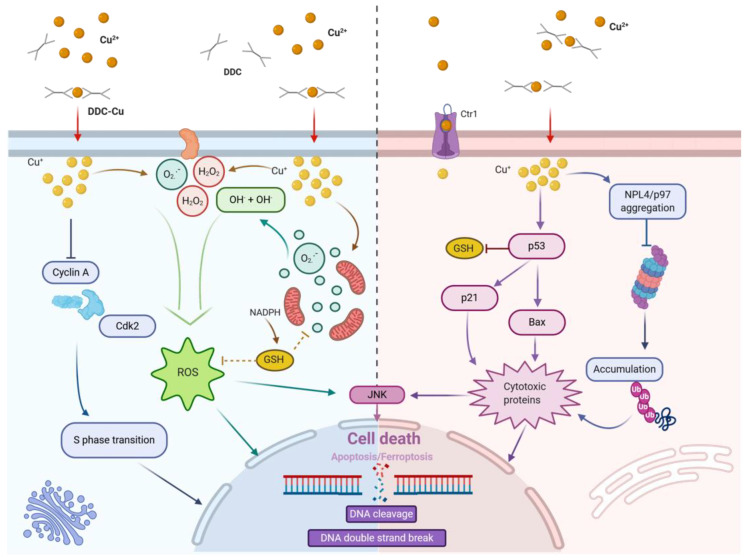
Mode of action of disulfiram-derived diethyldihtiocarbamate (DDC) on cancer cells. Cellular uptake of DDC-Cu^2+^ causes an increase in the free copper pool (Cu^+^) which provokes massive induction of reactive oxygen species (ROS) that have a vast range of effects, including the induction of DNA damage (**left**). At the same time, the copper accumulation leads to the activation of p53 signalling, dysfunction of proteasomal removal of poly-ubiquitinylated proteins, activation of stress-activated protein kinases like JNK and the induction of death pathways (**right**).

The high sensitivity of tumor cells to DSF in general and melanoma cells in particular can be explained by their inherently increased levels of reactive oxygen species (ROS) and a high number of free radicals [21]. In transformed melanoma cells, metal-bound melanin is responsible for increased ROS formation, which stimulates tumor cell growth. [18,19,21,23,24,25]. Although increased ROS levels were shown to have a mitogenic potential, they also resulted in a reduced ability to deal with additional oxidative stress compared to melanocytes [21]. In line with this, melanoma cells were found to contain higher Cu^2+^ concentrations [19,23], which enhanced the cytotoxic effects of DSF [9,18,19]. When cells were treated with DSF and bathocuproine disulfonic acid (BCPD), a cell-membrane-impermeable copper ion chelator, intracellular copper levels did not increase, and the anti-proliferative and cytotoxic effects were reversed [18,22,23]. In summary, the cytotoxic effects and the increased ROS production induced by DSF were found to be largely dependent on the presence and the amount of Cu^2+^ [18,23] (Figure 2). 

On the other hand, ROS renders the cells vulnerable to additional oxidative stress and facilitates accumulation of DNA-damage. DNA-damage is usually sensed by the cell and induces a DNA-damage response [19]. In case of heavy damage, this includes mitochondrial pore opening and apoptosis [21,22,25]. When DSF was added to cells, lipid ROS levels increased and in the presence of Cu^2+^ or Zn^2+^, ROS production was further augmented. The DSF mediated increase in intracellular copper and ROS levels was also seen in melanoma cells [19,23], suggesting that Cu^2+^ was bound in the extracellular space before shuttling into the cells [22]. 

Furthermore, DSF has been shown to inhibit superoxide dismutase 1 (SOD1) in melanoma cells by complexing Cu^2+^ in a way that SOD1 lacks Cu^2+^ for its activity [19]. This can lead to increased formation of superoxide and oxidative stress [19]. The increased ROS levels were found to be essential for the induction of apoptosis by DSF in melanoma cells [18]. Vice versa, the ROS scavenger n-acetyl cysteine (NAC) prevented PARP cleavage and apoptosis induction by DSF plus Cu^2+^ treatment [18]. 

Further, it was observed that DSF competes with reduced glutathione (GSH) for glutathione reductase (GSR), causing an imbalance in glutathione homeostasis. The inhibition of the enzyme GSR blocked the GSH redox cycling, which led to an accumulation of oxidized glutathione (GSSG) and a lower GSH/GSSG ratio [21,25]. DSF also affected the mitochondrial membrane polarization by oxidizing thiol groups of mitochondrial proteins. This increased the mitochondrial membrane permeability and induced pore opening [25]. Thiuram disulfides such as DSF are often metabolized in the mitochondria in an NAD(P)H- and GSH-dependent manner and can cause irreversible oxidation of GSH and NAD(P)H. Furthermore, swelling of the mitochondria and inhibition of oxidative phosphorylation could be observed. [26]. 

### 5.2. Disulfiram and Cell Death

The increased uptake of Cu^2+^ in DSF-treated melanoma cells leads to higher oxidative stress, which can trigger the extrinsic apoptosis pathway. [18]. Increased PARP and caspase 8 cleavage were identified as indicators for extrinsic apoptosis signalling but neither caspase 9 nor BID cleavage, which would be typical for the intrinsic apoptosis pathway, could be detected [18]. Furthermore, it was possible to block DSF activity and reduce apoptosis induction by treating the cells with a caspase 8 inhibitor that prevented caspase 8 and PARP cleavage [18]. 

Recently, it was found that after treatment of nasopharyngeal cancer cells, multiple myeloma cells or leukemia cells with DSF plus Cu^2+^, the protein level and phosphorylation of c-Jun expression were increased [16,27,28]. In chemotherapy-resistant HL60 leukemia cells, treatment with DSF plus Cu^2+^ led to a re-sensitization of the cells to doxorubicin and antracycline [28]. The JNK/c-Jun pathway is involved in the regulation of proliferation, differentiation and apoptosis and can be activated by pro-inflammatory cytokines, anticancer drugs or environmental stress, like UV radiation [27,28]. The activated JNK/c-Jun pathway can then induce pro-apoptotic proteins like p53, Bax and Fas. Kamata et al. discovered that ROS, which was caused by tumor necrosis factor alpha (TNF-α), further induced a sustained JNK activation in mouse fibroblast. There, the catalytic cysteine of the JNK-inactivating phosphatase was converted into sulfenic acid [29]. They also found that ROS accumulation runs parallel to the induction of TNF-α-mediated programmed cell death. [29]. This supports the previously described role of ROS for the anti-cancer effects of DSF. Shi et al. confirmed the hypothesis of ROS-mediated JNK activation. They identified that sustained JNK activity modulates the phosphorylation of serines in the histone protein H2AX and p53, thereby inhibiting proto-oncogenes [30].

In line with this, Li et al. showed that DSF plus Cu^2+^ has antitumor activity against nasopharyngeal cancer through a p53-mediated ferroptosis pathway [16]. They found that the expression of p53 target genes, such as p21 and Bax, increased after treatment with DSF and Cu^2+^ and ferroptosis was induced [16].

Interestingly, DSF not only induces cell death and apoptosis, but has recently been described to block the formation of membrane pores by gasdermine D (GSDMD) [31]. The inhibition of pore formation by GSDMD is crucial in inflammatory processes. This is the key step in pyroptosis and in the release of cytokines like IL-1β or IL-8 during inflammation. The inhibition of gasdermin D-dependent pore formation by DSF was mediated via a covalent modification of cysteine 191/192 (human/mouse). The inhibitory effect of DSF on membrane pore formation by GSDMD could be measured, as demonstrated in several human cells (THP-1, HEK293T, HCT116 and HT-29) and mouse cells (iBMDM). However, DSF did not show a protective effect on necroptosis or apoptosis in this study [31].

### 5.3. Disulfiram and Proteasome Inhibition

In general, it is known that cancer cells are relatively sensitive to proteasome inhibition, especially when compared with benign cells. DSF was found to block POH1, which is important for the intrinsic de-ubiquitination activity of the proteasome [17]. The proteasome cleaves the inhibitor-κB (IκB) [32], and thereby releases the heterodimer p50/p65 from the inhibitory complex to translocate to the nucleus and regulate gene transcription [17,33]. For this reason, the activity of the proteasome is necessary for the activation of NF-κB and its anti-apoptotic, cancer-promoting and cell-cycle-regulating effects. [17,34]. If the proteasome is blocked by DSF, IκB continuously inhibits NF-κB and favours cancer cell death. Thus, the combination of DSF and Cu^2+^ blocked the proteasome and induced apoptosis specifically in prostate [35] and breast cancer cells, but not in healthy breast epithelial cells [36]. Further, the combination of DSF with Zn^2+^ potently inhibited the 20S proteasome [37] and showed anti-melanoma activity [38]. In summary, DSF negatively affects NF-κB activity by its capacity to block proteasome activity [17]. 

However, a recent publication showed that some proteasome-dependent NF-κB activity was present under DSF treatment, which suggests that DSF inhibits neither 20S nor 26S proteasome activity in a direct manner [9]. In the same manuscript, it was reported that CuET/DDC-Cu^2+^ treatment affected protein degradation upstream of the proteasome [9]. The p97 ATPase activity was unchanged but NPL4 became immobilized in focal clusters in the nucleus and the cytoplasm [9]. P97 and NPL4, together with UFD1, are key components of a complex with chaperone or segregase function for proteins that are targeted for proteasomal degradation and need to be released from binding partners or cellular structures like the endoplasmic reticulum (ER). NPL4 contains a Zn-finger domain with two zinc fingers. It is known that Zn-fingers bind bivalent metal ions, which probably induced the direct interaction between NPL4 and CuET. [9]. VCP/p97 interacts with the NPL4 aggregates and stays immobilized, leading to a disabled p97-NPL4-UFD1 pathway. This inhibition was shown to trigger a heat-shock response and ER stress, which further sustained the cell death pathways [9].

The same group also discovered that cells lacking BRCA1 and BRCA2 were particularly sensitive to DSF and Cu^2+^ (CuET) treatment [39]. CuET induced replication stress-associated DNA damage in several cancer cell lines and increased γH2AX, as an indicator for DNA double-strand breaks in these cells (DSB) [39]. Homologous recombination (HR), a DSB repair mechanism, requires BRCA1 and BRCA2 for its activity. Interestingly, a co-localization of ATR with immobilized NLP4 aggregates was found after CuET treatment. Moreover, CuET interfered with the activation of the RPA-ATRIP-ATR-CHK1 repair pathway, by suppressing the ATR kinase despite of high replication stress, induced by ssDNA aggregates [39]. 

### 5.4. Disulfiram and Transcription Factor Modifications

DSF was shown to form mixed sulphides with sulfhydryl containing transcription factors (TF) in melanoma cells [22,40]. Cysteines are often found in the DNA binding region of TF such as NF-κB or ATF/CREB [22,40]. Thus, DSF can block such transcription factors by binding to their DNA binding region [15,22,41] and mediating S-glutathionylation, which renders the TF negatively charged [22,40]. If metal ions such as Cu^2+^ or Zn^2+^ were present, the formation of mixed sulphides was further enhanced [22]. Glutathione can also react with mixed sulphides, which then become even more negatively charged, resulting in no DNA binding activity at all [22]. Thus, when DSF and Cu^2+^ are added to cells, TF-DNA binding was significantly reduced [22]. For example, cyclin A is positively regulated by CRE. When cells were treated with DSF and Cu^2+^, mixed sulphides formed between DSF and CRE, in such a way that cyclin A was downregulated. This resulted in reduced cell cycle progression into the G2-M phase [22,40]. DSF was also shown to form mixed sulphides with DNA topoisomerases and blocked cell proliferation already at the stage of DNA replication [42]. Other examples for mixed sulphides are the ATF/CREB TF complex, which is responsible for the proliferation and survival or NF-κB, which induces the expression of anti-apoptotic genes. 

### 5.5. Disulfiram and Cancer Cell Invasion

In lung and bladder tumor models treated with DSF plus Zn^2+^, more necrotic tissue was found compared to control tissues. Additionally, less tumor neovascularization was observed in SCID mice treated with DSF and Zn^2+^. Extracellular matrix (ECM) degradation by Zn^2+^-dependent matrix metalloproteases (MMP) plays a big role in cell invasion and angiogenesis. DSF showed type IV collagenase inhibitory activity, which was responsible for blocking invasion and angiogenesis. Similarly, DSF directly interacted with MMP-2 and MMP-9 and inhibited their proteolytic activity because of the chelation of the protease co-factor Zn^2+^ [43]. 

## 6. The Use of Disulfiram in Cancer Patients 

Edward Frederick Lewison, an American physician at Johns Hopkins Hospital, published the first clinical case report on DSF as an anti-cancer therapy in 1977. He described a 35-year-old woman with metastatic breast cancer who had to discontinue her hormone therapy because of her severe alcohol syndrome and was replaced by DSF. Surprisingly, after starting the DSF therapy for alcohol withdrawal, she showed spontaneous cancer regression with complete remission of all metastases in the spine, skull, pelvis and ribs. From 1961 to 1971, the patient received no further cancer therapy. In 1971, however, she tragically died as a result of a third-floor window fall [44]. This case revealed the potential cancer-inhibiting effect of DSF and opened the door to several experimental research projects and clinical trials in various oncological fields (Table 1 and Table 2). 

### 6.1. Clinical Trials

The majority of the initiated trials were early phase (I/II) studies with a small number of recruited patients and a variety of solid cancers. The main outcome of the studies with published results [44,46,47,48,49,50,51] was an acceptable tolerability with common grade 1 and 2 toxicities, including fatigue, dysgeusia, nausea, vomiting, diarrhea, ataxia, renal failure and LFT elevations in up to 100% of the enrolled patients [48]. The most tolerable dose for DSF was approximately 250 mg/day, with a maximum tolerated dose of 500 mg/day [49]. In contrast to the case report mentioned above, the trials with DSF as monotherapy, even when supplemented with copper, did not show clinical efficacy in terms of response according to RECIST (Table 1). This could be due to the fact that the biological availability of DSF and its metabolite diethyldithiocarbamate in the circulatory system is insufficient due to an unfavorable pharmacology. However, clinical benefits such as disease stabilization or improvement in overall or progression-free survival were particularly evident in the combination approaches of DSF with chemotherapeutic agents [46,49,50,51]. To date, no clinical data have been published where DSF is combined with modern targeted therapies or immune-oncology therapies like immune-checkpoint blockade (Table 2). 

### 6.2. Epidemiological Data

Skrott et al. [9] investigated whether the use of DSF has an impact on cancer mortality by analyzing the Danish national demographic and health registers. Cancer-specific mortality was higher among former DSF users than among never-DSF users, most likely due to the link between alcohol abuse and cancer. Most interestingly, they identified a lower cancer-specific mortality for cancer overall and specifically for colorectal, prostate and breast cancer among post-cancer persistent DSF users compared to previous DSF users who discontinued DSF therapy. This advantage of the DSF continuation group even translated into improved hazard ratios for cancer-specific mortality in patients with metastatic disease [9]. In a previous analysis conducted in the same Danish registry, 53,856 disulfiram users were reported to have developed 166, 644 and 464 cases of melanoma, breast or prostate cancer. The odds ratios were calculated for cases of melanoma, breast or prostate cancer associated with long-term disulfiram use. The results indicated a protective effect of DSF against prostate and breast cancer, but not against melanoma [15]. Although these epidemiological results do not permit conclusions on causality, they support the hypothesis that DSF may have anticancer effects.

## 7. Drug Combinations with Disulfiram

DSF was studied for several years as a new therapy in oncology [10,21] and its potential as an anti-cancer drug was widely described. Additionally, its activity as an adjuvant therapeutic in combination with other cancer therapies has been reported in recent years. Numerous in vitro and in vivo experiments confirmed that DSF significantly enhances the cytotoxic effects of anticancer therapies such as chemotherapy and radiotherapy [10,52,53,54,55,56] (Table 3).

### 7.1. Combinations of Disulfiram with Chemotherapy 

DSF can serve as a complementary anticancer therapy to chemotherapeutic drugs such as oxaliplatin and temozolomide (TMZ), providing an additive cytotoxic effect [57,58]. Calderon-Aparicio et al. demonstrated that DSF enhances oxaliplatin uptake of colorectal cancer cells SW620 (*KRAS* and *TP53* mutated), resulting in higher DNA damage [58]. There, DSF significantly increased the cytotoxic effect of oxaliplatin in SW620 colorectal cancer cells in an additive manner [58]. Triscott et al. [57] reported that DSF downregulated the expression of Polo-like kinase 1 (PLK 1) and prevented the development of resistance to temozolomide (TMZ) in BT74 and GBM4 glioblastoma cells. PLK1 is related to a proliferative and aggressive subtype of GBM, which has the highest rate of resistance to TMZ and the worst prognosis. As a result of the downregulation of PLK1 by DSF, the growth of the primary glioblastoma cell lines BT74 and GBM4 was diminished [57]. In addition, DSF, in combination with 1-(2-chloroethyl)-1-nitrosourea (BCNU), strongly inhibited MGMT (O6-methylguanine-DNA methyltransferase) in vitro and in in T98 glioblastoma xenografts. MGMT is a DNA repair protein which eliminates the O (6)-alkyl groups in guanines, thereby providing resistance to alkylating therapies in glioblastoma cells [59]. Moreover, the pre-incubation with DSF enhanced the cytotoxic effect of BCNU on U87 glioblastoma cells by a factor of three. Simultaneously, the G2/M blockade performed by BCNU was improved [59]. Wang et al. [54] showed that DSF, in combination with 5-fluorouracil (5-FU), substantially increased the cytotoxic effect of 5-FU on DLD-1 and RKO colon carcinoma cell lines. In addition, DSF decreased 5-FU chemo-resistance in the 5-FU-resistant cell line H630 by the inhibition of NF-kappa B activity [54].

The potential of DSF in combination with cisplatin was also investigated. Bodenner et al. [60] demonstrated that DSF efficiently reduces cisplatin-induced bone marrow toxicity and nephrotoxicity in B6D2F_1_ mice, F344 rats and a beagle dog model. In addition, DSF at doses of 250-300 mg acted as an antiemetic in the beagle dog model. Interestingly, this chemo-protective effect did not interfere with the anti-cancer therapeutic effect in the B16 melanoma and Lewis lung carcinoma models in B6D2F1 mice [60]. 

Additionally, DSF was shown to block the P-glycoprotein/ MDR1 efflux transporter, which is extruding chemotherapeutics from malignant cells. The MDR1-blocking mechanism of DSF is possibly a cysteine modification, which renders the cells more sensitive to xenobiotics [22,40,61]. 

### 7.2. Combination of Disulfiram with Radiotherapy

Radiotherapy has been one of the main treatment modalities for solid tumors in recent years. According to several studies, DSF significantly increased radio-sensitivity in neuroblastoma, glioblastoma and breast cancer models [52,53,54]. Rae et al. proved that DSF acts as a radio-sensitizer in neuroblastoma cells through the massive generation of oxidative stress, in addition to the inhibition of proteasomal activity [53]. Further, DSF was found to enhance the toxicity of γ- and iodine-131 –meta-iodobenzylguanidine (131/I-MIBG) radiation against neuroblastoma in a copper-dependent manner. This enhancement proved to be synergistic [53]. Similarly, Kang-Koh et al. established that DSF improves radio-sensitivity in glioblastoma cells. This effect was predominantly observed in glioblastoma cells that had an unmethylated O6-methylguanine-DNA methyltransferase promoter (MGMT-WT). Such cells are sturdily resistant to radiotherapy. DSF triggered radio-sensitization by inhibiting the repair of radiation-induced DNA damage, resulting in G2/M arrest and apoptosis induction. Interestingly, DSF did not mediate radio-sensitization in glioblastoma cells with a methylated MGMT promoter [52]. Wang et al. tested DSF in combination with radiotherapy in breast cancer models. The combination significantly inhibited the growth of MDA-MB-231-luc-D3H1 and 4T1 tumors and their associated lung metastases in the BALB/c mice. Mechanistically, the inhibition of NF-κB could be detected [54]. Due to the accumulating evidence, DSF is a promising drug for combination with radiotherapy 

### 7.3. Combination of Disulfiram with ROS Inducers

Several other therapies were combined with DSF to test their combinatorial anti-cancer effects. A combination therapy of DSF and auranofin, a gold-comprising composite used as a therapy for rheumatoid diseases, was described. Auranofin acts as a sturdy inhibitor of the proteasome-associated deubiquitinases (DUBs), producing a high accumulation of intracellular ubiquitinated protein and reactive oxygen species (ROS). Huang et al. [55] showed a synergistic cytotoxicity effect of auranofin in combination with DSF in hepatocellular carcinoma models in vitro and in vivo (mice xenografts). This synergistic effect was achieved by a rapid induction of endoplasmic reticulum stress and an immense intracellular ROS production, with subsequent caspase activation and apoptosis induction [55]. DSF was also combined with arsenic trioxide (As_2_O_3_), a pentavalent semi-metal used to treat acute promyelocytic leukemia. As_2_O_3_ abruptly decreased the glutathione levels in melanoma cells, obstructing the ROS scavenging system. Hence, DSF in combination with As_2_O_3_ enhanced the blocking of the ROS scavenger mechanism and amplified intracellular ROS production, yielding strong apoptosis induction [21]. Subsequently, Trapp et al. tested the superoxide dismutase inhibitor tetrathiomolybdate (ATN-224) in combination with DSF [24]. ATN-224 is an intracellular Cu^2+^ chelator and did diminish DSF-induced ROS production. Therefore, the interaction between ATN-224 and DSF was antagonistic [24]. These data strongly support the concept of the dependence of DDC-Cu^2+^ on the production of ROS [21,24,55].

### 7.4. Combination of Disulfiram with Targeted Therapies

DSF was further studied as a combined therapy with target therapy, particularly with MEK inhibitors. Calderon-Aparicio et al. screened in A375 and C8161 melanoma cells in vitro for an effective combination drug with MEK inhibitors (GSK1120212). DSF in combination with the MEK inhibitor UO126 showed a synergistic cytotoxic effect on melanoma cells. The effects could be antagonized by the addition of ammonium tetrathiomolybdate (TTM), due to its copper chelating potential [58]. This outcome was explained by the chelation and sequestering of available copper, which is an essential co-factor for MEK1/2 in the oncogenic MAPK pathway signaling [58,62]. Cu^2+^ influx is known to increase MEK1 activity [62,63] due to the capacity of MEK1 to bind two copper ions, which favors the interaction with and phosphorylation of ERK1/2 [62,64]. Vice versa, MEK1 activity was found to be reduced in the absence of copper, [58,63], even in the context of a BRAF mutation or a constitutive activation of RAS/RAF signaling [62]. 

Additionally, Fernandez et al. described that DSF, in combination with GSK1120212 (MEK inhibitor), had an additive effect in terms of growth inhibition on transformed melanocytic cells in the zebrafish model [65]. Zhao et al. conducted various experiments with the multi-kinase inhibitor regorafenib co-delivered with disulfiram/copper. Regorafenib significantly blocked glioma cell proliferation and re-educated tumor-associated macrophages (TAMs) towards an anti-tumor TAM1 polarization [56]. 

### 7.5. Combination of Disulfiram with Immunotherapy

There is accumulating evidence that the tumor micro-environment, including the TAMs, significantly influence tumor progression and response to immunotherapy. Therefore, new combination strategies combining immune-checkpoint blockade with immune modulating drugs were developed. In 2020, Terashima et al. demonstrated that DSF enhances the immune-checkpoint (PD-1 antibody) therapy responses in a lung cancer and melanoma model [66]. DSF with anti-PD-1 antibody blockade showed a significant additive effect in the Lewis lung carcinoma model and an extensive synergistic effect in the B16 melanoma model. Mechanistically, DSF induced the inhibition of FROUNT, a cytoplasmatic protein that interacts with CCR5 and CCR2 and co-activates the PI3K-Rac-lamellipodium pathway. Simultaneously, the number of granzyme B- and CD8-positive, tumor-infiltrating T cells increased. This strongly supported the idea that DSF could enhance the anti-PD-1-based immune-checkpoint blockade [66].

## 8. Conclusions

In many experimental in vitro studies, disulfiram and its metabolite diethyldithiocarbamate have been shown to be highly effective in inhibiting tumor cell proliferation and in inducing tumor cell death. This applies to various types of cancer, including malignant melanoma of the skin. However, no reliably measurable efficacy of disulfiram as a mono-therapeutic agent against solid tumors has been observed in clinical trials to date, maybe due to the lack of biological availability in the circulating system. Because of the promising and complex mechanisms of disulfiram, which can lead to the death of tumor cells, it is still a promising approach to provide additional benefit to cancer and melanoma patients, in combination with other therapeutic modalities such as chemo-, radio-, targeted- and immunotherapy. Further experimental investigations and the results of the ongoing clinical combination studies will shed light on this. Disulfiram may, therefore, evolve from a myth to a new therapeutic approach for malignant melanoma, for which there is still a high clinical need. This is especially true for patients who show no benefit after standard of care, become resistant or belong to somatic mutation subgroups for which no targeted therapy option exists, such as BRAF wild-type melanomas including NRAS mutant and NF1 loss-of-function melanomas.

## Figures and Tables

**Table 1:** Clinical trials using disulfiram as monotherapy in cancer patients.

Phase	Title	Status	Study Results	Cancer	Drug (Monotherapy)	Trial ID & Ref.
I	Phase I Study of Disulfiram and Copper Gluconate for the Treatment of Refractory Solid Tumors Involving the Liver	Completed	Well tolerated; no dose limiting toxicity; one stable disease	Cancer	Disulfiram Copper Gluconate	NCT00742911 [43]
I	Bioavailability of Disulfiram and Metformin in Glioblastomas	Recruiting	N/A	Glioblastoma	Disulfiram	NCT03151772
Ib	A Phase Ib Study of Intravenous Copper Loading with Oral Disulfiram in Metastatic, Castration Resistant Prostate Cancer	Terminated	No grade > 3 toxicities; no effect on PSA; 64Cu-PET shows Cu-uptake in some metastases	Prostate Cancer	Disulfiram Copper gluconate	NCT02963051 [45]
I/II	Disulfiram in Patients with Metastatic Melanoma	Completed	N/A	Stage IV Melanoma	Disulfiram	NCT00256230
II	Study of Recurrent Prostate Cancer with Rising Prostate Specific Antigen (PSA)	Completed	Moderate tolerability (6/19 with grade 3); 5/19 (26%) patients with change in 5-methyl-cytosine; no effect on PSA levels	Prostate Cancer	Disulfiram	NCT01118741 [46]
II	Phase II Trial of Disulfiram with Copper in Metastatic Breast Cancer	Recruiting	N/A	Metastatic Breast Cancer	Disulfiram Copper	NCT03323346
II	Disulfiram and Cisplatin in Refractory TGCTs.	Recruiting	N/A	Germ Cell Tumor	Disulfiram	NCT03950830

**Table 2:** Clinical trials using disulfiram as combination therapy in cancer patients.

Phase	Title	Status	Study Results	Cancer	Drug (Combination Therapy)	Trial ID & Ref.
I	Disulfiram Plus Arsenic Trioxide in Patients with Metastatic Melanoma and at Least One Prior Systemic Therapy	Terminated	N/A	Metastatic Melanoma	Disulfiram + Arsenic trioxide	NCT00571116
I	Disulfiram in Treating Patients with Glioblastoma Multiforme After Radiation Therapy with Temozolomide	Completed	Max. tolerated dose 500 mg/day	Glioblastoma	Temozolomide Disulfiram Copper gluconate	NCT01907165 [44]
I	Disulfiram and Gemcitabine Hydrochloride in Treating Patients with Unresectable Solid Tumors or Metastatic Pancreatic Cancer	Recruiting	N/A	Stage IV Pancreatic Cancer	Disulfiram + Gemcitabine Hydrochloride	NCT02671890
I	Phase 1 study of Disulfiram and Nivolumab for gastric cancer	Recruiting	N/A	Gastric cancer	Disulfiram + Nivolumab	jRCTs0311883
I/II	Disulfiram/Copper with Concurrent Radiation Therapy and Temozolomide in Patients with Newly Diagnosed Glioblastoma	Recruiting	Low toxicity at 250 mg/day. Median follow-up of 12.3 months, 1-year PFS: 57%; 1-year OS: 69%.	Glioblastoma Multiforme	Disulfiram Copper Gluconate Surgery/ Radiation Temozolomide	NCT02715609 [47]
I/II	A Proof-of-concept Clinical Trial Assessing 9 Repurposed Drugs Combined with Metronomic Temozolomide (CUSP9v3 Treatment Protocol) for Recurrent Glioblastoma	Active, not recruiting	N/A	Glioblastoma	Temozolomide + Disulfiram	NCT02770378
II	Disulfiram/Copper Combination in The Treatment of Newly Diagnosed Glioblastoma Multiforme	Unknown status	N/A	Glioblastoma Multiforme	Temozolomide Disulfiram Copper	NCT01777919
II	Disulfiram and Chelated Zinc for the Rx of Disseminated Mets Mel That Has Failed First Line Therapy	Completed	1 grade 3+ toxicity (confusional episode); ORR 0/12; 1 target lesion –27%	Melanoma	Disulfiram and chelated zinc	NCT02101008 [48]
II	Safety, Tolerability and Efficacy of Disulfiram and Copper Gluconate in Recurrent Glioblastoma	Completed	Low toxicity (1/23 with grade 3 elevated ALT); ORR 0/23; 14% with clinical benefit	Recurrent Glio-blastoma	Disulfiram/Copper Temozolomide (TMZ)	NCT03034135 [49]
II	Disulfiram and Copper Gluconate with Temozolomide in Unmethylated Glioblastoma Multiforme	Recruiting	N/A	Glio-blastoma Multiforme	Disulfiram Copper gluconate Temozolomide	NCT03363659
II	Disulfiram-Copper Gluconate in Met Pancreas Cancer w Rising CA19-9 on Abraxane-Gemzar, FOLFIRINOX or Gemcitabine	Recruiting	N/A	Metastatic Pancreatic Cancer	Disulfiram Copper + Nab-Paclitaxel/Gemcitabine, Gemcitabine or FOLFIRINOX	NCT03714555
II	Vinorelbine, Cisplatin, Disulfiram and Copper in CTC_EMT Positive Refractory Metastatic Breast Cancer.	Recruiting	N/A	Metastatic Breast Cancer	Vinorelbine, Cisplatin, Disulfiram and Copper	NCT04265274
IIb	Initial Assessment of the Effect of the Addition of Disulfiram (Antabuse) to Standard Chemotherapy in Lung Cancer	Completed	Benefit in PFS (5.9 vs. 4.9 mo) and OS (10.0 vs. 7.1 mo)	Non-small Cell Lung Cancer	Chemotherapy ± disulfiram	NCT00312819 [50]
II/III	Disulfiram in Recurrent Glioblastoma	Active, not recruiting	N/A	Glio-blastoma	Disulfiram Copper Alkylating Agents	NCT02678975

**Table 3:** Disulfiram and drug/therapy combinations.

Therapy	Description	Cancer Type and Model	Effect of Combination
Temozolomide (TMZ)	Imidazo-tetrazine derived alkylating chemotherapy	Glioblastoma in vitro: BT74, GBM4 and short-term cultures	Inhibtion of chemoresistance development
Oxaliplatin	Platinum-based chemotherapy	Colorectal cancer in vitro: SW-620^KRAS G12V^ p53 mutation	Additive cytotoxic effect to the Oxaliplatin
BCNU (1,3-bis-2- chloroethyl- nitrosourea)	MGMT (O6-methylguanine methyltransferase)- alkylating agent	Human glioblastoma in vitro: U87 and T98G	The preincubation with 50 µM DSF for 12 h enhance 3-fold the cytotoxicity effect of BCNU compared with BCNU alone.
5-fluorouracil (5-FU)	Antineoplastic agent	Colorectal cancer in vitro: DLD-1, RKO_WT_	Enhancement of the 5-FU cytotoxicity
Cisplatin (Cl_2_H_6_N_2_Pt)	Platinum-based chemotherapy	Melanoma Lung carcinoma Colon carcinoma in vivo: L1210 and P388 leukemia B16 melanoma Lewis lung Colon 26 in B6D2F_1_ (C57BL/6xDBA/2F_1_) mice	Provision of a chemoprotective effect
Radio therapy	High energy doses of radiation	Glioblastoma in vitro: U138MG, T98G, U251MG, U87M and U373MG	Enhancement of radio-sensitivity
Radio therapy	High energy doses of radiation	Neuroblastoma Glioblastoma in vitro: SK-N-BE(2c), UVW-NAT in vivo: SK-N-BE(2c), UVW-NAT in CD-1 nu/nu mice	Induction of radio-sensitization
Radio therapy	High energy doses of radiation	Breast cancer in vitro: MDA-MB-231, *MDA*-MB-231-luc-D3H1, SUM149, UACC-812 in vivo: MDA-MB-231-luc in NOD/SCID mice 4T1 cells in BALB/c mice	Reduction of tumor growth and lung metastases formation
Auranofin	Gold salt	Hepatocellular carcinoma in vitro: HepG2 and SMMC-7721 in vivo: HepG2 and SMMC-7721 in BALB/c nude mice	Enhancement of Auranofin-induced apoptosis
Tetra-thio molybdate (ATN-224)	Superoxide dismutase inhibitor	Melanoma in vitro: M14 and YUZAZ6	Antagonistic effect
Arsenic trioxide (As_2_O_3_)	A pentavalent semimetal	Melanoma In vitro: A375	Increase of ROS production
UO126	MEK1/2 inhibitor	Melanoma in vitro: C8161^KRASmut^, A375^BRAFmut^	Synergistic improvement of the tumor suppression effect of MEK inhibition.
GSK 1120212 (Trametinib), PD184352	MEK1/2 inhibitor	Melanoma in vitro: WM852^NRASmut^, D04^NRASmut^, A375^BRAFmut^, 501Mel^BRAFmut^, WM266-4^BRAFmut^ in vivo: V12RAS zebrafish	Additive cytotoxic effect in combination with MEK inhibitors.
Regorafenib	Macrophage modulator	Glioblastoma in vitro: U87 and GL261 in vivo: U87 in BALB/c mice GL261 in C57BL/6 mice	Synergistic effect in the conversion and polarization of the TUMs, leading to an increased antitumoral effect.
Clone J43, BioXcell	Antibody against the immune-checkpoint PD-1	Melanoma Lung carcinoma in vitro: Lewis lung carcinoma (LLC), B16F10 (B16), THP-1 cells, and CHO cells. 16-DsRed and LLC-DsRed (For visualization) in vivo: *B16F10* and LLC in C57BL/6 mice	FROUNT inhibition and thereby decrease of tumor progression and TAMs activity
